# Non-valvular Atrial Fibrillation in CKD: Role of Vitamin K Antagonists and Direct Oral Anticoagulants. A Narrative Review

**DOI:** 10.3389/fmed.2021.654620

**Published:** 2021-09-17

**Authors:** Aleix Cases, Pablo Gomez, Jose Jesus Broseta, Elisa Perez Bernat, Juan de Dios Arjona Barrionuevo, Jose Maria Portolés, Jose Luis Gorriz

**Affiliations:** ^1^Departament de Medicina, Universitat de Barcelona, Institut d'Investigacions Biomèdiques August Pi i Sunyer (IDIBAPS), Barcelona, Spain; ^2^Unidad de Factores de Riesgo Vascular, Servicio de Nefrología, Hospital Universitario de Jerez, Jerez de la Frontera, Spain; ^3^Departament de Medicina, Universitat de Valencia, Valencia, Spain; ^4^Hospital Clínico Universitario de Valencia, Valencia, Spain; ^5^Servicio de Cardiología, Hospital Universitario Virgen del Rocío, Sevilla, Spain; ^6^Hospital Universitario Puerta de Hierro, Madrid, Spain; ^7^Instituto de Investigación del Hospital Clinico Universitario, Valencia (INCLIVA), Valencia, Spain

**Keywords:** chronic kidney disease, atrial fibrillation, Direct oral anticoagulants, Vitamin K antagonists, anticoagulant-related nephropathy

## Abstract

Atrial fibrillation (AF) is the most common arrhythmia in chronic kidney disease (CKD), with a close bidirectional relationship between the two entities. The presence of CKD in AF increases the risk of thromboembolic events, mortality and bleeding. Vitamin K antagonists (VKA) have been the mainstay of treatment for the prevention of thromboembolic events in AF until recently, with confirmed benefits in AF patients with stage 3 CKD. However, the risk-benefit profile of VKA in patients with AF and stages 4–5 CKD is controversial due to the lack of evidence from randomized controlled trials. Treatment with VKA in CKD patients has been associated with conditions such as poorer anticoagulation quality, increased risk of bleeding, faster progression of vascular/valvular calcification and higher risk of calciphylaxis. Direct oral anticoagulants (DOACs) have shown equal or greater efficacy in stroke/systemic embolism prevention, and a better safety profile than VKA in *post-hoc* analysis of the pivotal randomized controlled trials in patients with non-valvular AF and stage 3 CKD, yet evidence of its risk-benefit profile in more advanced stages of CKD is scarce. Observational studies associate DOACs with a good safety/effectiveness profile compared to VKA in non-dialysis CKD patients. Further, DOACs have been associated with a lower risk of acute kidney injury and CKD development/progression than VKA. This narrative review summarizes the evidence of the efficacy and safety of warfarin and DOACs in patients with AF at different CKD stages, as well as their effects on renal function, vascular/valvular calcification and bone health.

## Introduction

Atrial fibrillation (AF) is the most common cardiac arrhythmia and is associated with an increased risk of stroke or systemic embolism (S/SE) (1). Moreover, AF-related stroke is usually more severe, associated with greater disability, increased mortality and longer hospital stays (2).

Chronic kidney disease (CKD) is also common, with increasing prevalence worldwide (3, 4). Atrial fibrillation is more prevalent in CKD, in both non-dialysis (ND) (16–21% and increasing with higher CKD stages), and particularly end-stage kidney disease (ESKD) patients on dialysis (17–27%) (5). In fact, the relationship between AF and CKD is bidirectional: AF is associated with an increased risk of CKD [reduced glomerular filtration rate (GFR) or albuminuria], while CKD is linked to increased incidence/prevalence of AF (6–9). Atrial fibrillation and CKD share several risks factors (older age, hypertension, diabetes, prevalent cardiovascular disease); while inflammation, frequent in CKD, has been involved in initiation/perpetuation of AF (10). Patients with severe CKD frequently have cardiac abnormalities favoring AF development (left ventricular hypertrophy, myocardial fibrosis or left atrial enlargement) (11). Furthermore, rapid shifts in serum pH and electrolytes during hemodialysis (HD) sessions may be a trigger for AF.

Oral anticoagulation therapy (OAT) with vitamin K antagonists (VKA) and more recently direct oral anticoagulants (DOACs) have been the cornerstone of S/SE prevention in patients with non-valvular AF (NVAF) at risk for stroke. However, the presence of CKD in AF further heightens the risk of S/SE (46–49% in ND-CKD or 83% in ESKD) and death (60–65% higher risk in CKD or ESKD) (5), as well as risk of bleeding in patients under OAT, raising doubts about the risk-benefit profile (5, 9, 12). Atrial fibrillation is associated with a prothrombotic state through numerous pathophysiological pathways, which is further aggravated by CKD, via changes in the left atrium, endothelial dysfunction or activation of coagulation and platelets (9). However, there is no clear evidence of a significant increase in S/SE risk in ESKD patients with AF. Possible explanations for this include: the high prevalence/incidence of non-AF-related stroke in ESKD, uremic platelet dysfunction, use of intradialytic heparin and the paroxysmal and self-limiting nature of hemodialysis-associated AF bursts, which should be associated with a low risk of S/SE, in contrast to the persistent/permanent AF associated with chronic structural atrial changes (13, 14). Thus, if no association between AF and S/SE in hemodialysis patients was proven, there would not be a need for OAT in this population (13).

## Vitamin K Antagonists in Patients With AF and CKD

Vitamin K antagonists reduce S/SE incidence in patients with NVAF in the general population (15). However, VKA have several drawbacks: slow onset of action and cessation of effects, a narrow therapeutic range requiring frequent monitoring and dose adjustments, and significant drug-drug and food-drug interactions (16), which may be aggravated by dietary restrictions in CKD (17).

Evidence of the efficacy and safety of VKA in CKD patients with AF from randomized controlled trials (RCT) is scarce. Warfarin targeted at an international normalized ratio (INR) of 2–3 vs. aspirin plus fixed low-dose of warfarin, reduced the risk of S/SE by 76% without increasing the risk of major bleeding in the AF patient subset with stage 3 CKD (18). However, there is no RCT evidence of the risk/benefit ratio of VKA in patients with AF and stage ≥ stage 4 CKD.

In a meta-analysis of observational studies, warfarin reduced the risk of S/SE and mortality (30 and 35%, respectively) without increasing the risk of major bleeding in the ND-CKD group; in ESKD patients, however, warfarin did not decrease the risk of S/SE or mortality, yet increased the risk of major bleeding by 30%, suggesting that VKAs have a favorable risk/benefit profile in ND-CKD patients, but not in ESKD (19). This agrees with an updated meta-analysis of 15 observational studies in ESKD patients with AF, in which those receiving warfarin, had a 49% increased rate of hemorrhagic stroke with no benefits on ischemic stroke (IS), major bleeding or mortality vs. those not receiving warfarin (20), and also with the results of two recent studies; an observational study (21) and a retrospective study of patients with advanced CKD and incident AF before initiating dialysis (22). Despite the limitations of these studies (observational, results subject to confounding, moderate to high heterogeneity, no data on quality of anticoagulation), they suggest that VKA may not be effective for S/SE prevention in patients with AF and ESKD, and indeed may be harmful (20, 23). In addition, the time in therapeutic range (TTR) is lower in CKD patients; the worse the renal function, the lower the TTR; while poor anticoagulation quality is associated with increased risk of thromboembolic and bleeding events (24–26). INR variability has also been associated with bleeding and mortality in ESKD patients (27). Further, patients with moderate-severe CKD usually require lower doses of VKA, and show a higher bleeding risk with supra-therapeutic INR (28, 29). Further information on the net clinical benefit of VKA in this population is forthcoming from two ongoing RCTs comparing the hemorrhagic and thrombotic risk of VKA vs. no anticoagulation in hemodialysis patients with AF: the Oral Anticoagulation in Hemodialysis Patients (AVKDIAL) (NCT02886962), and the Danish Warfarin-Dialysis Study - Safety and Efficacy of Warfarin in Patients With Atrial Fibrillation on Dialysis (DANWARD) (NCT03862859).

## Direct Oral Anticoagulants in Patients With AF and CKD

The efficacy of DOACs (dabigatran, rivaroxaban, apixaban and edoxaban) in S/SE prevention and their safety profile vs. VKA have been demonstrated in pivotal trials (30–33). A meta-analysis of these trials found that DOACs have greater efficacy in reducing S/SE, mainly driven by reducing hemorrhagic stroke, lowering the risk of mortality, and conferring a non-significant lower risk of major bleeding (significant for intracranial hemorrhage) (34).

However, DOAC rely on the kidney, to variable extent, for drug elimination. Renal clearance is about 80% with dabigatran, 50% with edoxaban, 33% with rivaroxaban and 27% with apixaban (35), and these may require dose adjustments in moderate-severe CKD (Table 1). In this regard, it could be useful to measure the anticoagulant effect of DOACs in advanced CKD or in patients with deteriorating renal function (36).

**Table 1:** DOAC pharmacological properties and dose adjustments by renal function.

	**Dabigatran**	**Apixaban**	**Rivaroxaban**	**Edoxaban**
Bioavailability	3–7%	50%	66–100 %	62%
Protein binding	35%	87%	>90%	55%
Half-life	12–14 h	~12 h	5–9 h (young) 11–13 h (elderly)	10–14 h
Renal elimination	>80%	27%	33%	50%
Dializability	Yes (50–60%)	Small	No	No
Reversal agent	Idarucizumab	Andexanet alfa	Andexanet alfa	Andexanet alfa
Usual dose	150 mg bid or 110 mg bid (Europe)	5 mg bid	20 mg/d	60 mg/d
**Dose adjustments by renal function**				
Europe	CrCl 30–50 110 mg bid if high bleeding risk CrCl < 30 Contraindicated	CrCl> 30: Sr Cr ≥ 1.5 mg/dl and 1 of 2 (age ≥ 80 yrs or weight ≤ 60 kg) 2.5 mg bid, otherwise 5 mg bid CrCl ≤ 30 2.5 mg bid CrCl < 15 Contraindicated	CrCl 15–50 15 mg/d CrCl< 15 Contraindicated	CrCl 15–50 30 mg/d CrCl < 15 Contraindicated
United States	CrCl > 30 150 mg bid CrCl 15–30 75 mg bid CrCl< 15 Contraindicated	At least 2/3, Sr Cr ≥ 1.5 mg/dl, age ≥ 80 yrs, weight ≤ 60 kg 2.5 mg bid, otherwise 5 mg bid ESKD 5 mg bid (age < 80 yrs or weight > 60 kg)	CrCl 15–50 15 mg/d CrCl < 15 or dialysis 15 mg/d (limited data)	CrCl > 95 Contraindicated CrCl 15–50 30 mg/d CrCl < 15 Contraindicated

*bid, twice daily; ESKD, End-stage kidney disease; CrCl, Creatinine clearance; Sr Cr, Serum creatinine*.

Among patients with moderate CKD *post-hoc* analysis of pivotal RCTs of DOACs vs. warfarin in patients with NVAF (37–41), showed a favorable risk-benefit profile for DOACs, which has been confirmed in several meta-analyses (42–44). In the Cochrane meta-analysis including 12,545 AF patients with CKD (390 patients in stage 4), DOACs reduced the incidence of S/SE (RR 0.81, 95% CI 0.65–1.00) and non-significantly reduced the incidence of major bleeding (RR 0.79, 95% CI 0.59–1.04) vs. warfarin (42). However, these studies included patients with creatinine clearances (CrCl) > 30 ml/min [the Apixaban for the Prevention of Stroke in Subjects with Atrial Fibrillation (ARISTOTLE) trial included patients with CrCl > 25 ml/min]. Therefore, the risk/benefit profile of DOACs in patients with AF and stages 4–5D CKD is uncertain. A *post-hoc* analysis of the ARISTOTLE trial confirmed the safety profile of apixaban with a 66% lower risk of major bleeding among patients with CrCl 25–30 ml/min (45).

Several observational studies have evaluated the effectiveness and safety of DOACs vs. warfarin, covering a range of different CKD stages (26, 46–62) (see Table 2). Despite the low quality of the evidence, due to heterogeneity or retrospective study designs, they suggest that DOACs are a viable alternative to VKA in patients with ND-CKD, with a net clinical benefit observed in a meta-analysis (69).

**Table 2:** Observational studies comparing DOACs vs. VKA or no anticoagulation in CKD patients.

**References**	**Study design**	**Patients**	**DOACs type**	**Comparator**	**Efficacy outcomes (HR, 95% CI)** ^*^	**Safety outcomes (HR, 95% CI)** ^*^
Lee et al. (46)	Retrospective	eGFR < 60 ml/min per 1.73 m^2^	Dabigatran Rivaroxaban (*n* = 59)	VKA (*n* = 174)	Stroke (HR 0.78, 0.21–3.00) Composite of death, hospitalization and stroke (HR 0.3, 0.11–0.77) No differences in mortality	Major bleeding (HR 0.23, 0.07–0.69)
Harel et al. (47)	Population-based Nested case-control study	Stages 3–4 CKD, aged ≥ 66 yrs	Dabigatran (*n* = 200) Rivaroxaban (*n* = 33)	VKA (*n* = 8,176)		Major bleeding Dabigatran [adjusted odds ratio (aOR), 0.9, 0.64–1.25] Rivaroxaban (aOR, 0.97, 0.44–2.11) vs. warfarin
Stanton et al. (48)	Retrospective Matched cohort	CrCl < 25 ml/min or ESKD (AF 72.6%)	Apixaban (*n* = 73)	VKA (*n* = 73)	Stroke (7.5 vs. 7.5%)	Major bleeding (9.6 vs. 17.8%, *p* = 0.149)
Weir et al. (49)	Retrospective IPTW	CKD (CrCl < 50 ml/min)	Rivaroxaban (*n* = 229)	VKA (*n* = 647)	IS (HR 0.09, 0.01–0.72) Composite event (VTE, MI, IS) (HR 0.56; 0.26–1.18)	Major bleeding (HR 1.20, 0.66–2.20)
Shin et al. (55)	Retrospective Propensity- score matched cohort	eGFR < 60 ml/min per 1.73 m^2^	Dabigatran Rivaroxaban Apixaban (*n* = 1,122)	VKA (*n* = 1,122)	IS (HR 1.02, 0.76–1.37)	Bleeding (HR 1.23, 1.02–1.48)
Di Lullo et al. (56)	Retrospective	Stages 3b-4 CKD	Rivaroxaban (*n* = 247)	VKA (*n* = 100)	All strokes (warfarin 25 in 24 patients vs. 0 with rivaroxaban *p* ≤ 0.002)	GI bleeding (8 warfarin vs 2 rivaroxaban, *p* < 0.001)
Becattini et al. (50)	Prospective	Stages 1–4 CKD (*n* = 449)	Dabigatran Rivaroxaban Apixaban	Stable vs. worsening renal function (WRF)		Major bleeding (HR 1.02, 1.01–1.04) for every 1 mL min^−1^ decrease in eGFR (HR 2.43, 1.33–4.45) for WRF with change in eGFR staging
Kumar et al. (51)	Population-based Retrospective Propensity score	Incident AF, CKD (eGFR < 50 ml/min per 1.73 m^2^) and ≥ 65 yrs, (patients on dialysis excluded)	OAT (*n* = 2424) DOACs or VKA	No OAT (*n* = 2424)	IS (HR 2.60, 2.00–3.38) Mortality (HR 0.82, 0.74–0.91)	Bleeding (HR 2.42, 1.44–4.05)
Schafer et al. (52)	Retrospective	Stages 4–5 CKD or dialysis (NVAF or VTE)	Apixaban (*n* = 302)	VKA (*n* = 302)	S/SE similar for both groups in each of the time periods	Bleeding 0–3 months (OR 0.58; CI 0.31–1.11) 6–12 months (OR 0.16; 0.05–0.5)
Loo et al. (57)	Population-based Retrospective Propensity score	Stages 3–5 CKD, dialysis and KTR and incident AF	Rivaroxaban Apixaban Dabigatran Edoxaban (*n* = 2,596)	VKA (*n* = 2,596)	IS/SE (HR 0.79, 0.40–1.58)	Major bleeding (HR 0.88, 0.47–1.62) GI bleeding (HR 0.99; 0.63–1.55)
Bonnemeier et al. (54)	Retrospective database Propensity score	New users of OAT and CKD	Rivaroxaban (*n* = 4,164)	VKA (*n* = 7,002)	IS (HR 0.72, 0.55–0.94)	ICH (HR 0.66, 0.38–1.14)
Coleman et al. (58)	Retrospective	Stages 4–5 CKD (88% stage 5 or hemodialysis)	Rivaroxaban (*n* = 1,986)	VKA (*n* = 4,848)	S/SE (HR 0.55, 0.27–1.10) IS (HR 0.67, 0.30–1.50)	Major bleeding: (HR 0.68, 0.47–0.99)
Wetmore et al. (53)	Retrospective IPTW	Stages 3–5 CKD (not on dialysis) and incident AF	Apixaban (*n* = 6,738) Rivaroxaban (*n* = 3,904) Dabigatran (*n* = 1,588)	VKA (*n* = 10,529)	S/SE (HR 0.70, 0.51–0.96 for apixaban) (HR 0.80, 0.54–1.17 for rivaroxaban) (HR 1.15, 0.69–1.94 for dabigatran) No differences in mortality	Major bleeding (HR 0.47, 0.37–0.59 for apixaban) (HR 1.05, 0.85–1.30 for rivaroxaban) (HR 0.95, 0.70–1.31 for dabigatran)
Chan et al. (60)	Retrospective	Stages 4–5 CKD (25% on dialysis)	DOACs (*n* = 280) VKA (*n* = 520)	No OAT (*n* = 2,971)	IS/SE Warfarin [adjusted hazard ratio (aHR) 3.1, 2.1–4.6] vs. no OAT DOAC (aHR 1.1; 0.3–3.4) vs. no OAT	Major bleeding Warfarin (aHR 2.8; 2.0–3.8) DOACs (aHR 3.1, 1.9–5.2).
Weir et al. (26)	Retrospective Propensity score	Stages 4–5 CKD and dialysis	Rivaroxaban (*n* = 781)	VKA (*n* = 1536)	IS/SE (HR 0.93, 0.46–1.9)	Major bleeding (HR 0.91, 0.65–1.28)
Yao et al. (61)	Retrospective US adiministrative claims database Stabilized IPTW	New users of OAT, eGFR ≥ 15 ml/min per 1.73 m^2^	Apixaban (*n* = 10,880) Dabigatran (*n* = 3,007) Rivaroxaban (*n* = 8,269)	VKA (*n* = 10,680)	Apixaban: Stroke (HR 0.57, 0.43–0.75), Mortality (HR 0.68, 0.56–0.83); Dabigatran: Stroke, (HR, 0.94, 0.65–1.35) Mortality (HR, 0.68, 0.48–0.98); Rivaroxaban: Stroke (HR, 0.69, 0.51–0.94), Mortality (HR, 0.73, 0.58–0.91). No interaction between treatment and eGFR for any outcome	Major bleeding Apixaban (HR 0.51, 0.44–0.61) Dabigatran (HR 0.57, 0.43–0.75) Rivaroxaban (HR 0.84, 0.72–0.99)
Ashley et al. (62)	Retrospective Population-level High-dimensional propensity core	Incident patients, ≥ 66 years, starting OAT of all stages of CKD	DOACs (*n* = 27552) (dabigatran 32%, rivaroxaban 41%, apixaban 27%)	VKA (*n* = 27552)	Cardiovascular event or mortality (HR 0.82, 0.75, 0.90) Interaction eGFR and primary outcome by OAT class (*p* = 0.02) eGFR ≥ 60 (HR 1.01, 0.92–1.12), eGFR 30–59 (HR 0.83, 0.75–0.93), eGFR < 30 (HR 0.75, 0.51, 1.10)	Bleeding (HR 0.73, 0.58, 0.91) No interaction
Chan et al. (63)	Retrospective	Hemodialysis patients Fresenius Medical Care North America ESKD database (*n* = 29,977)	Rivaroxaban (*n* = 244) Dabigatran (*n* = 281)	VKA (*n* = 8,064) Aspirin (*n* = 6,018)		Hospitalization or death from bleeding vs. warfarin Dabigatran (RR 1.48; 1.21–1.81) Rivaroxaban (RR 1.38, 1.03–1.83) Hemorrhagic death: Dabigatran (RR, 1.78; 1.18–2.68) Rivaroxaban (RR, 1.71; 0.94–3.12)
Sarrat et al. (64)	Retrospective In center	Hemodialysis patients	Apixaban (*n* = 40)	VKA (*n* = 120)		Major bleeding (0 vs. 5.8%, *p* = NS)
Siontis et al. (65)	Retrospective cohort, Propensity score	Dialysis patients of Medicare	Apixaban (*n* = 2,351)	VKA (*n* = 7,053)	S/SE (HR 0.88, 0.69–1.12) Mortality (HR, 0.85; 0.71–1.01)	Major bleeding (HR 0.72, 0.59–0.87) Intracraneal bleeding (HR, 0.79; 0.49–1.26)
Mavrakanas et al. (66)	Retrospective US Renal Data System cohort Propensity score	Dialysis patients with incident AF	Apixaban (*n* = 521)	No treatment (*n* = 1561)	Stroke, TIA, SE (HR 1.24, 0.69–2.23) Mortality (HR, 0.58; 0.43–0.78)	Fatal /IC bleeding (HR 2.74, 1.37–5.47)
Makani et al. (59)	Retrospective	Stages 3–5 CKD or dialysis patients	Dabigatran Rivaroxaban Apixaban Edoxaban (*n* = 4,748)	VKA (*n* = 5,895)	Embolic stroke Stage 3 CKD (HR 0.87, 0.73–1.04) Stage 4–5 CKD (HR 0.60, 0.34–1.09) Mortality Stage 3 CKD (HR 0.74, 0.68–0.81) Stage 4–5 CKD (HR 0.76, 0.63–0.92)	Bleeding Stage 3 CKD (HR 0.83, 0.74–0.94) Stage 4–5 CKD (HR 0.69, 0.50–0.93)
See et al. (67)	Retrospective Population-based cohort Propensity score	Dialysis patients with NVAF	DOACS (*n* = 448)	Warfarin (*n* = 448)	IS/SE (HR 1.21, 0.76–1.92)	ICH (HR 0.78, 0.29–2.1) Major bleeding (HR 0.98; (0.64–1.51))
See et al. (67)	Retrospective Population- based cohort Propensity score	Dialysis patients with NVAF	OAT (*n* = 2,977)	No OAT (*n* = 2,977)	IS/SE (HR 1.54, 1.29–1.84)	Major bleeding (HR 1.14, 0.97–1.34) ICH (HR 1.41, 0.99–2.02)
Miao et al. (68)	Retrospective IPTW	ESKD or on dialysis patients	Rivaroxaban (*n* = 787)	Apixaban (*n* = 1,836)	S/SE (HR 1.18, 0.53–2.63) IS (HR 1.12, 0.45–2.76)	Major bleeding (HR 1.00, 0.63–1.58)

* *Unless otherwise stated. CKD, Chronic kidney disease; CrCl, Creatinine clearance; DOACs, Direct oral anticoagulants; ESKD, End-stage kidney disease; GI, Gastrointestinal; HR, Hazard ratio; ICH, Intracerebral hemorrhage; IPTW, Inverse-probability-of-treatment weighting approach; IS, Ischemic stroke; KTR, Kidney transplant recipient; MI, Myocardial infarction; NVAF, Non-valvular atrial fibrillation; OAT, Oral anticoagulation therapy; SE, Systemic embolism; USRDS, US Renal Data System; VKA, Vitamin K antagonists; VTE, Venous thromboembolism*.

In ESKD patients observational studies comparing DOACs vs. VKA show high heterogenicity (63–68, 70), with initial studies showing safety concerns with dabigatran or rivaroxaban vs. warfarin (63). In contrast, a retrospective study comparing apixaban vs. warfarin showed similar event rates for IS/SE but, significantly lower rates of major bleeding events. Furthermore, 5 mg/12 h apixaban was associated with lower risk for IS/SE and mortality vs. warfarin (65). However, recent studies have not confirmed these benefits (67), or the benefit of DOACs vs. non-OAT in this population (66).

A RCT in hemodialysis patients with NVAF compared the effect of VKA, rivaroxaban at 10 mg/d and rivaroxaban at 10 mg/d plus vitamin K2 at 2,000 μg/thrice weekly for 18 months on the progression of cardio-aortic calcium deposits (primary endpoint). After the initial follow-up, only severe bleeding events were lower in rivaroxaban-treated patients (71). However, after a follow-up extension of the trial for at least 18 additional months, a reduction was observed in the composite outcome of fatal and non-fatal cardiovascular events and of life-threatening or major bleeding complications compared with VKA, although the risk of stroke or death were not different between groups (72).

In a recent meta-analysis in ESKD patients with AF, DOACs showed comparable effectiveness and safety to VKA, while OAT vs. no anticoagulation was associated with a higher risk of IS/SE and a similar risk of major bleeding (67). Thus, there is no evidence of a net clinical benefit of DOACs in ESKD patients with AF, despite an urgent need. Several ongoing RCT are assessing OAT use and comparing the safety and efficacy of DOACs vs. VKAs in this population [Compare Apixaban and vitamin-K antagonists in patients with AF and ESKD (AXADIA); NCT02933697] [Strategies for the Management of Atrial Fibrillation in patiEnts Receiving Dialysis (SAFE-D); NCT03987711] which will likely yield useful data.

## Control of Renal Function During Oral Anticoagulation

Worsening of kidney function (WRF) is common among AF patients (73, 74) and is associated with an increased risk of cardiovascular events, all-cause mortality and bleeding (41, 73–75). This worsening is also common in observational studies (50, 76, 77), especially among CKD patients, and is a better predictor of IS/SE and bleeding than renal dysfunction *per se* (50, 78). Together with changes in renal function over time because of concomitant treatments (e.g., RAAS blockade and diuretics) or acute events, WRF during follow-up requires monitoring to adjust DOAC dosage. Discordance in DOAC prescription with drug labeling is common in clinical practice, and may result in overdosing or underdosing. Overdosing of DOAC due to inadequate adjustment increases the risk of bleeding, while underdosing is associated with an increased risk of stroke, especially with apixaban (79, 80).

Dose adjustment of DOAC according to drug labeling is based on estimated CrCl using the Cockroft-Gault (CG) formula, while laboratories usually report eGFR (MDRD or CKD-EPI equations), which provide a better estimation of measured GFR, especially for bed-ridden patients whose weight is difficult to estimate (81, 82). Discrepancies in results between different equations may result in inappropriate dosing and patient misclassification (e.g., as eligible for DOACs when they are contraindicated) (83–86). This issue will likely be addressed in the near future, but until more data are available it is reasonable to use the CG-eCrCl formula to adjust DOAC dose.

Guidelines recommend measuring renal function at DOAC treatment initiation and annually thereafter, but suggest more frequent monitoring in CKD patients > 75 years-old, with frailty, and those treated with DOAC with higher renal clearance (87). In CKD patients the interval for monitoring kidney function (in months) can be calculated by dividing eGFR/10. Monitoring renal function in patients under DOAC treatment should also be considered when WRF is suspected, in contexts such as acute events or when prescribing drugs that affect renal hemodynamics (87).

## Complications During OAT: DOACs vs. VKA

### Effects on Kidney Function

#### Acute Kidney Injury/Anticoagulant-Related Nephropathy

Warfarin-related nephropathy (WRN) is an episode of acute kidney injury (AKI) associated with warfarin and was first reported in 2009 (88). It is characterized by an increase in serum creatinine, with micro or macrohematuria, in the absence of other causes of AKI, often following supra-therapeutic INR values. Subsequent studies have reported further cases of WRN, most associated with an INR > 3 (89). Kidney biopsies showed signs of acute tubular injury and glomerular hemorrhage with red blood cells in Bowman's space and renal tubular obstruction by red blood cell casts. Although all cases had underlying renal disease in the initial study (88), later studies reported WRN in both CKD and non-CKD patients, and it was also associated with increased mortality risk (89) and faster CKD progression (90).

Several cases have also been reported of AKI in patients treated with DOACs, particularly with dabigatran. Pathological findings were similar to those found in WRN, and this entity is now known as anticoagulant-related nephropathy (ARN). In a recent histologic study of ARN most cases had underlying renal disease (91), suggesting that excessive anticoagulation aggravates an underlying glomerular disease and increases glomerular hematuria. Other possible mechanisms of AKI during OAT include oxidative stress due to free iron release and hemoglobin tubular toxicity.

The risk of AKI in patients with AF is high. In an observational study, one in seven had an AKI episode within 2 years (76). The risk of this injury was higher among patients with CKD and those receiving VKA vs. DOAC-treated patients in several observational studies (76, 92–95).

#### CKD Development and Progression

*Post-hoc* analysis of *the Rivaroxaban Once Daily Oral Direct Factor Xa Inhibition Compared with Vitamin K Antagonism for Prevention of Stroke and Embolism Trial in Atrial Fibrillation* (ROCKET) (73) and *Randomized Evaluation of Long-Term Anticoagulant Therapy with Dabigatran etexilate* (RE-LY) (96) trials suggest that DOACs have a more beneficial effect on renal function decline than warfarin, which has been confirmed in multiple observational studies (76, 94, 95, 97–99). The renal benefit of DOACs was more apparent with rivaroxaban and dabigatran, and was notably better than in VKA-treated patients with supra-therapeutic INR levels.

The renal benefit of DOACs vs. VKA may be owing to its more predictable dose-response effect and lower risk of hemorrhages; the lower risk of AKI which could influence progressive deterioration of renal function, and its potential renal anti-inflammatory effect, as observed in experimental models (100). In contrast, VKA can promote vascular calcification (as discussed below), increasing arterial stiffness, favoring systolic hypertension and a pulsatile flow at the microvasculature, which may be deleterious for the kidney (101).

Lastly, the *Factor XA Inhibition in REnal Patients with Non-valvular Atrial Fibrillation-Observational Registry* (XARENO) (NCT02663076) is an observational prospective study that will compare CKD progression with rivaroxaban, VKA or no anticoagulation in patients with CKD stages 3–4 and NVAF.

### Vascular and Valvular Calcification and Risk of Calciphylaxis

A common complication in CKD patients, vascular calcification (VC) is inversely associated with renal function and positively associated with worse prognosis in this population (102). Further, vascular medial calcification favors arterial stiffness, which is associated with systolic hypertension, left ventricular hypertrophy and reduced coronary flow reserve, and is a risk factor for cardiovascular events, and CKD progression (101). Vitamin K antagonists impede the -carboxylation of coagulation factors, as well as other vitamin K-dependent proteins which inhibit VC, such as matrix Gla protein, Gla-rich protein, and growth arrest–specific protein 6, preventing their activation (103).

In fact, VKA have been associated with increased progression of VC (104), and arterial stiffness (105), which may be especially relevant in CKD patients, and supporting that DOACs are safer than VKA as regards these two conditions, although the differential effect was not demonstrated in a recent RCT (71). Although switching from warfarin to rivaroxaban improved arterial stiffness in a prospective study (106), this finding was not substantiated in the above mentioned RCT (71). Further, rivaroxaban was also found to reduce the progression of aortic or mitral valve calcification vs. VKA in an observational, retrospective study in stage 3b-4 CKD patients (107).

Calciphylaxis is a rare but severe complication in dialysis patients, characterized by skin necrosis and ulceration, with panniculitis, calcification and luminal occlusion of small arterioles and subcutaneous capillaries (108, 109). Calciphylaxis is associated with high morbidity and mortality. Treatment with VKA increases several fold the risk of calciphylaxis in both uremic and non-uremic patients (110), suggesting a beneficial effect for DOACs in preventing this complication.

### OAT and Bone Health

Patients with ND-CKD and ESKD are prone to bone fractures and osteoporosis due to factors such as bone mineral disease (111). Vitamin K antagonists inhibit the γ-carboxylation of osteocalcin which plays an important role in bone matrix formation (17). Nonetheless, the role of VKA on bone fractures and bone density is controversial. In a recent meta-analysis, VKA for >1 year was not associated with increased risk of fracture vs. controls or compared to DOACs, although the risk was increased in females and elderly patients vs. controls (112).

The available evidence suggests that DOACs are associated with a lower impact on bone metabolism and potentially with lower risk of fractures than VKA (113–120), although this warrants corroboration in properly designed studies. Interestingly, switching from warfarin to rivaroxaban was associated with an improvement in bone metabolism markers (106).

## Left Atrial Appendage Occlusion

Given the increased risk of bleeding among patients with severe CKD, left atrial appendage occlusion (LAAO) may be an attractive alternative to reduce the risk of S/SE events while avoiding the need for OAT. All the RCTs showing LAAO as non-inferior to OAT in preventing stroke and bleeding events had excluded patients with severe CKD; however a recent prospective study showed that, at 2 years' follow-up, LAAO in dialysis patients was associated with no thromboembolic events and lower bleeding incidence compared to a cohort treated with oral anticoagulants (121), similar results were found in a small study (122). Besides this study, a prospective single arm study of the Watchman device in dialysis patients is currently ongoing (NCT03446794).

## Key Concepts

1. Atrial fibrillation prevalence and incidence is higher in CKD and rises with the degree of kidney dysfunction. The presence of CKD in AF confers an increased risk of stroke/systemic embolism, mortality, and bleeding.
2. Oral anticoagulation with VKA in mild-moderate CKD patients with AF results in a net clinical benefit, although there are no conclusive data on their risk/benefit ratio in more advanced stages of CKD, especially among ESKD patients. Vitamin K antagonist treatment in CKD is associated with poorer anticoagulation quality and increased risk of bleeding.
3. Pivotal RCTs have demonstrated a net clinical benefit for DOACs vs. warfarin in NVAF with mild-moderate CKD, but there is little evidence in patients with AF and stages ≥ 4 CKD. Observational studies suggest that the benefit remains in ND-CKD patients, but is controversial in ESKD.
4. Vitamin K antagonists are associated with a higher risk of anticoagulant-related nephropathy, acute kidney injury and faster CKD progression than DOACs, especially among CKD patients.
5. In patients with NVAF, worsening renal function is common and associated with poorer outcomes. Renal function should be periodically monitored and DOAC dose adjusted to avoid the risk of over or underdosing. Renal function should be measured using the CG-eCrCl, rather than eGFR.
6. Vitamin K antagonists have been associated with an increased risk of vascular/valvular calcification in CKD and likely with higher risk of osteoporosis/fractures, which may be particularly relevant in CKD.

## Author Contributions

AC and PG conceived and drafted the manuscript. AC, PG, JB, and EP carried out the literature search. JA, JP, and JG provided a critical revision of the manuscript. All authors contributed to the article and approved the final version of the manuscript.

## Conflict of Interest

The authors declare that the research was conducted in the absence of any commercial or financial relationships that could be construed as a potential conflict of interest.

## Publisher's Note

All claims expressed in this article are solely those of the authors and do not necessarily represent those of their affiliated organizations, or those of the publisher, the editors and the reviewers. Any product that may be evaluated in this article, or claim that may be made by its manufacturer, is not guaranteed or endorsed by the publisher.
